# Role of Environmental Chemicals in Diabetes and Obesity: A National Toxicology Program Workshop Review

**DOI:** 10.1289/ehp.1104597

**Published:** 2012-02-01

**Authors:** Kristina A. Thayer, Jerrold J. Heindel, John R. Bucher, Michael A. Gallo

**Affiliations:** 1 Division of the National Toxicology Program,; 2 Division of Extramural Research, Cellular, Organs and Systems Pathobiology Branch, Office of Health Assessment and Translation, and; 3 Division of the National Toxicology Program, National Institute of Environmental Health Sciences, National Institutes of Health, Department of Health and Human Services, Research Triangle Park, North Carolina, USA; 4 Department of Environmental and Occupational Medicine, Environmental and Occupational Health Sciences Institute, UMDNJ–Robert Wood Johnson Medical School, Piscataway, New Jersey, USA

**Keywords:** animal, diabetes, environment, epidemiology, glucose, insulin, *in vitro*, metabolic syndrome, obesity, pollution

## Abstract

Background: There has been increasing interest in the concept that exposures to environmental chemicals may be contributing factors to the epidemics of diabetes and obesity. On 11–13 January 2011, the National Institute of Environmental Health Sciences (NIEHS) Division of the National Toxicology Program (NTP) organized a workshop to evaluate the current state of the science on these topics of increasing public health concern.

Objective: The main objective of the workshop was to develop recommendations for a research agenda after completing a critical analysis of the literature for humans and experimental animals exposed to certain environmental chemicals. The environmental exposures considered at the workshop were arsenic, persistent organic pollutants, maternal smoking/nicotine, organotins, phthalates, bisphenol A, and pesticides. High-throughput screening data from Toxicology in the 21st Century (Tox21) were also considered as a way to evaluate potential cellular pathways and generate -hypotheses for testing which and how certain chemicals might perturb biological processes related to diabetes and obesity.

Conclusions: Overall, the review of the existing literature identified linkages between several of the environmental exposures and type 2 diabetes. There was also support for the “developmental obesogen” hypothesis, which suggests that chemical exposures may increase the risk of obesity by altering the differentiation of adipocytes or the development of neural circuits that regulate feeding behavior. The effects may be most apparent when the developmental exposure is combined with consumption of a high-calorie, high-carbohydrate, or high-fat diet later in life. Research on environmental chemical exposures and type 1 diabetes was very limited. This lack of research was considered a critical data gap. In this workshop review, we outline the major themes that emerged from the workshop and discuss activities that NIEHS/NTP is undertaking to address research recommendations. This review also serves as an introduction to an upcoming series of articles that review the literature regarding specific exposures and outcomes in more detail.

The current prevalence of diabetes and obesity is unprecedented in the United States and abroad. Based on data from 2005–2008, 25.6 million, or 11.3% of all people in the United States ≥ 20 years of age, have diagnosed or undiagnosed diabetes [Centers for Disease Control and Prevention (CDC) 2011]. The total direct medical costs and indirect costs (disability, work loss, premature death) associated with diabetes in the United States during 2007 was $174 billion (CDC 2011). Another 35% of people in this age category are estimated to have pre-diabetes, a condition where blood glucose is higher than normal but not high enough to be classified as diabetes. This condition is a predictor for the development of diabetes. Approximately 11% of people with pre-diabetes developed type 2 diabetes each year during the average 3 years of follow-up in the Diabetes Prevention Program, a major clinical trial conducted to assess intervention strategies to prevent or delay the onset of diabetes in people with impaired glucose tolerance (American Diabetes Association 2011; Knowler et al. 2002). Overweight and obesity are well-known risk factors for the development of type 2 diabetes, perhaps contributing to approximately 70% of cases (Eyre et al. 2004). The prevalence of obesity worldwide had doubled since 1980 (World Health Organization 2011). In the United States, the prevalence of obesity among children and adolescents 2–19 years of age has almost tripled since 1980, and it is estimated that 16.9%, or 12.5 million, are obese (Ogden and Carroll 2010). This trend is also apparent in preschool children 2–5 years of age, where obesity increased from 5% in 1976–1980 to 10.4% in 2007–2008 (Ogden and Carroll 2010). Similarly, increased body weights have also been reported in pets and laboratory animals over the past decades (Klimentidis et al. 2010).

Excess caloric consumption and a seden-tary lifestyle are well-recognized risk factors for obes-ity and diabetes. However, there is growing interest in the contribution of “non-traditional” risk factors (e.g., environ-mental chemicals, stress, micro-nutrients, gut microbiome) to the etiology of these health conditions. Research addressing the role of environmental chemicals in diabetes and obesity has rapidly expanded in the past several years. The White House Task Force on Childhood Obesity (2010), the National Institutes of Health (NIH 2011), and the National Institute of Diabetes and Digestive and Kidney Diseases (2011) all acknowledge the growing science base in this area and cite the need for research to improve understanding of the role of environmental exposures in order to facilitate future prevention strategies. To help develop such a research strategy, the National Institute of Environmental Health Sciences (NIEHS) Division of the National Toxicology Program (NTP) organized a state-of-the-science workshop in January 2011 titled “Role of Environmental Chemicals in the Development of Diabetes and Obesity” to evaluate the literature for evidence of associations between certain chemicals and risk of diabetes and/or obesity (NTP 2011b). The specific environmental exposures evaluated were arsenic, maternal smoking during pregnancy/nicotine, organic tin compounds (“organotins”), phthalates, bisphenol A (BPA), pesticides, and various persistent organic pollutants (POPs). A diverse group of more than 50 scientists including endocrinologists, toxicologists, epidemiologists, bioinformaticists, and experts in the pathobiology of diabetes and obesity were asked to evaluate the current literature for consistency and biological plausi-bility, with the ultimate goal of providing advice to NIEHS for developing a research agenda on these emerging topics. Literature review documents, meeting presentations, and other background materials for the workshop are available online (NTP 2011b).

Overall, the existing literature was judged to provide plausibility, varying from suggestive to strong, that exposure to environmental chemicals may contribute to the epidemic of diabetes and/or obesity. This workshop review provides an overview of the major themes emerging from the workshop and describes several activities that NIEHS is undertaking to address research recommendations. This review also serves as the announcement of an upcoming series of papers to be published in *Environmental Health Perspectives* describing in more detail the critical assessment of the literature provided by the workshop participants.

## Methods

*Workshop format.* The workshop format was an introductory plenary session and a series of breakout group meetings, followed by plenary sessions to disseminate and discuss the findings from individual breakout group deliberations. A series of white papers was distributed before the workshop to help focus discussion. Breakout groups were not required to reach consensus on responses to charge questions, and plenary reports were prepared to reflect the range of opinions expressed. For the individual chemicals or chemical classes, workshop participants were asked to *a*) evaluate the strength/weaknesses, consistency, and biological plausibility of findings reported in humans and experimental animals; *b*) identify the most useful and relevant end points in experimental animals, *in vitro* models, and screening systems to assess these diseases; and *c*) identify data gaps and areas for future evaluation/research. Data from the Toxicology in the 21st Century (Tox21) High-Throughput Screening (HTS) Initiative were also considered during the meeting. Experts used the data, primarily derived from phase I of the U.S. Environmental Protection Agency (EPA) ToxCast™ (U.S. EPA 2011a), to help evaluate biological plausibility as well as to develop testable predictions of which chemicals might perturb biological processes related to diabetes and obesity. Experts were also asked to suggest relevant assay targets that could be included in Tox21 in the future to better screen for perturbations of these biological processes. Obesity is a major risk factor for metabolic syndrome and type 2 diabetes. All three outcomes were reviewed in relation to the environmental exposures evaluated during the workshop, although the primary focus and context varied for specific exposures.

*Literature search strategy.* A PubMed (National Library of Medicine, Bethesda, MD) search strategy was developed to identify studies of xenobiotic exposures related to diabetes and obesity using both a MeSH (Medical Subject Headings)-based strategy and a keyword strategy [for a complete list of MeSH and keyword search terms, see Supplemental Material (http://dx.doi.org/10.1289/ehp.1104597)]. The keyword search was included to identify newer articles that were not yet MeSH indexed in PubMed at the time of the search. Additional details about the criteria used to determine study rele-vance will be presented in subsequent publications that focus on specific exposures.

*Data extraction.* Data extraction of the main findings from studies considered relevant was conducted by NTP staff in the Office of Health Assessment and Translation (OHAT). Identification of main findings was based on the following strategy. For studies that did not report a significant association between the exposure and a health outcome, data extraction for the main finding was based on the highest exposure group compared with the referent group (e.g., fourth quartile vs. first quartile). When a study reported a significant association between an exposure and a health outcome, the data extraction for the main finding was based on lowest exposure group where a statistically significant association was observed and the shape of the exposure–response relation was monotonic (e.g., third quartile vs. first quartile). Identification of main findings when associations were non-monotonic in nature was conducted on a case-by-case basis and included consideration of any statistical trend analyses that might have been conducted, consistency of the overall pattern across exposure groups, and/or consideration of the author’s interpreta-tion of the biological significance of the non-monotonic finding.

An Excel file was used to store the data extraction output. This Excel file can be used in conjunction with a new graphical display software program called Meta Data Viewer developed by S. Harris at SRA International Inc. (Durham, NC, USA) and NTP OHAT staff (Boyles et al. 2011). In brief, the graphing program allows users to sort, group, or filter studies according to exposures, health outcomes, and other characteristics and can present the main findings using a “forest plot” graphical display. The input data file for the diabetes/obesity workshop contains approximately 870 main findings from > 200 human studies. This software program was used during the workshop to visually display data but was not used to conduct quanti-tative meta-analyses. The graphing program, accompanying data file, and instructions for use are publicly accessible (see NTP 2012; Boyles et al. 2011). Meta Data Viewer is a public resource, and users are welcome to use the program and any associated NTP data files for their own purposes, including for use in publications. Assistance in using the data file and software program is available upon request.

## Major Findings

**Figure 1:**
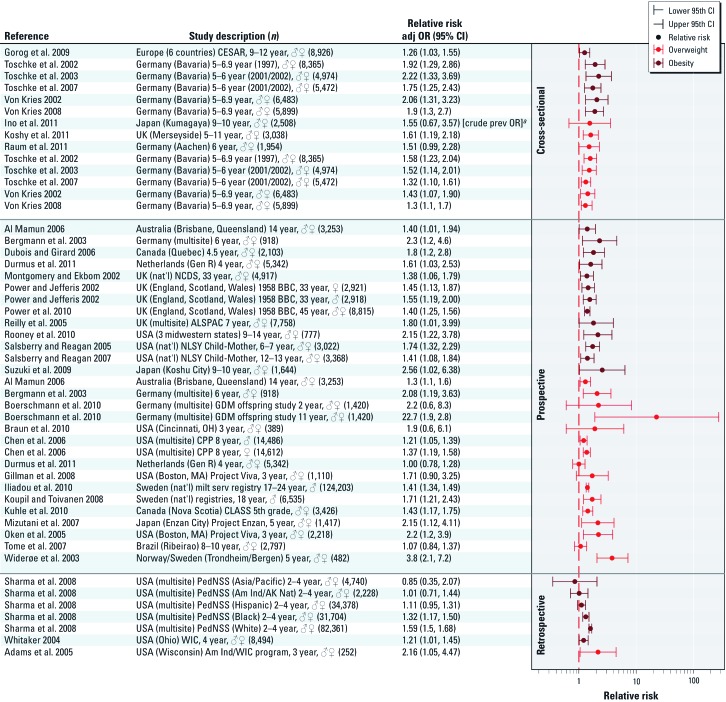
Association between maternal smoking during pregnancy and overweight/obesity in offspring. Studies are grouped by study design and then sorted by health outcome (overweight or obesity); studies are presented alphabetically by author within the health outcome categories. Abbreviations and symbols: ♀, female; ♂, male; AK Nat, Alaskan native; ALSPAC, Avon Longitudinal Study of Parents and Children; Am Ind, American Indian; adj OR, adjusted odds ratio; BBC, British Birth Cohort; CI, confidence interval; CESAR, Central European Study on Air Pollution and Respiratory Health; CLASS, Children’s Lifestyle and School Performance study; CPP, Collaborative Perinatal Project; GDM, gestational diabetes mellitus; Gen R, Generation R study; OH, Ohio; MA, Massachusetts; milt serv, military service; nat’l, national; NCDS, National Child Development Study; NLSY, National Longitudinal Survey of Youth; PedNSS, Pediatric Nutrition Surveillance System; prev OR, prevalence odds ratio; WI, Wisconsin; WIC, Women, Infants, and Children program. *^a^*Risk estimates for bracketed statistics (i.e., [crude prev OR]) calculated based on data presented in the paper using open source epidemiology statistics software OpenEpi (Dean et al. 2011).

*Maternal smoking and nicotine.* The strongest conclusion from the workshop was that nicotine likely acts as a developmental obesogen in humans. This conclusion was based on the very consistent pattern of overweight/obesity observed in epidemiology studies of children of mothers who smoked during pregnancy (Figure 1) and was supported by findings from laboratory animals exposed to nicotine during prenatal development. Crude and adjusted odds ratios (ORs) were similar within the individual epidemiological studies, suggesting that the social and behavioral characteristics that were included in models did not account for the observed differences in the prevalence of overweight (Oken et al. 2008). Two recent meta-analyses concluded there was some evidence for publication bias, but not enough to negate the overall conclusion of increased risk (Ino 2010; Oken et al. 2008). The body weight and adiposity-related changes reported in the animal studies recapitulated to a large extent those seen in children of mothers who smoke (Levin 2005; Newman et al. 1999; Oliveira et al. 2009, 2010a, 2010b; Santos-Silva et al. 2010, 2011; Somm et al. 2008; Williams and Kanagasabai 1984). The breakout group recognized that other components in cigarette smoke may also be contributing to the association between maternal smoking and childhood overweight/obesity; however, the studies of nicotine in experimental animals provided compelling evidence that nicotine alone was the causal agent.

**Figure 2:**
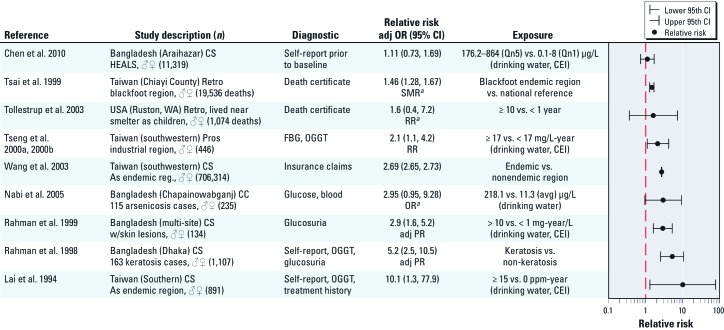
Association between arsenic and diabetes in areas of relatively high exposures (> 150 ppm drinking water). Studies are sorted by quality of the diagnostic from worse to better. Abbreviations: adj PR, adjusted prevalence ratio; As, arsenic; avg., average; CC, case–control; CEI, cumulative exposure index; CS, cross-sectional; FBG, fasting blood glucose; HEALS, Health Effects of Arsenic Longitudinal Study; OGTT, oral glucose tolerance test; Pros, prospective; Qn, quintile; Retro, retrospective; RR, relative risk; SMR, standardized mortality ratio; WA, Washington State. ***^a^***Calculated based on data presented using open source epidemiology statistics software OpenEpi (Dean et al. 2011) for Nabi et al. (2005) and Tsai et al. (1999) or as estimated by Navas-Acien et al. (2006) for Tollestrup et al. (2003).

*Arsenic.* The breakout group participants that evaluated this literature concluded that the existing human data were limited to sufficient in support of an association between arsenic and diabetes in populations with high exposure levels, namely, regions in Taiwan and Bangladesh with historical problems with arsenic contamination of drinking water (Figure 2). Although most members of the group considered the evidence sufficient for an association, additional research is needed to determine whether the relationship is causal. Workshop participants concluded that current evidence was insufficient for an association with diabetes and arsenic in lower--exposure areas (< 150 ppb in drinking water), such as the United States and Mexico, although recent studies with better measures of exposure and outcome provided increased evidence for an association (Coronado-Gonzalez et al. 2007; Del Razo et al. 2011; Ettinger 2009).

The literature on arsenic and diabetes in experimental animals was judged inconclusive. The body of existing studies is highly diverse, with considerable variation in the duration of treatment (1 day to 2 years), routes of adminis-tration, and dose levels used in the studies. Most of the studies treated animals with sodium arsenite [As(III); arsenic tri-oxide], but other arsenicals have also been studied (Aguilar et al. 1997; Arnold et al. 2003; Hill et al. 2009; Paul et al. 2008). The studies also vary in experimental design and model systems used to assess end points relevant to diabetes as a health effect. Most of the studies were not designed to examine the diabeto-genic effects of chronic arsenic exposure. Although the literature as a whole was judged inconclusive, findings from recent studies that were designed to focus more specifically on glucose homeostasis appear consistent with those human studies that link arsenic exposure to diabetes. Supportive findings include impaired glucose tolerance in studies of mice or rats treated with As(III) for several months at drinking water concentrations from 5 to 50 ppm (Cobo and Castineira 1997; Paul et al. 2007, 2008; Wang et al. 2009). In addition, measures of insulin regulation [e.g., homeo-static model assessment (HOMA) insulin resistance)] were affected in Wistar rats treated with 3.4 mg/kg body weight/day As(III) by oral gavage for 90 days (Izquierdo-Vega et al. 2006) and in pregnant female LM/Bc/Fnn mice treated with 9.6 mg/kg As(V) by intra-peritoneal injection on gestational days 7.5 and 8.5 (Hill et al. 2009).

Most *in vitro* or mechanistic studies were not designed specifically to study the diabetogenic or adipogenic effects of arsenic. Nevertheless, these studies suggest several pathways by which arsenic could influence pancreatic β-cell function and insulin sensitivity, including oxidative stress and effects on glucose uptake and transport, gluconeogenesis, adipocyte differentiation, and Ca^2+^ signaling (reviewed by Diaz-Villasenor et al. 2007, 2008; Druwe and Vaillancourt 2010; Tseng 2004). Studies suggest that arsenic may exert adverse effects on β-cell function *in vitro* through several mechanisms, depending on the concentration tested (Fu et al. 2010).

*Epidemiological studies of POPs and -diabetes.* POPs comprise a broad class of organohalides (i.e., organochlorines, organofluorines, and organobromines). The POP literature related to diabetes and other metabolic disorders is complex, consisting of approximately 75 epidemiological studies that report hundreds of findings relating to diabetes, altered glucose homeostasis, insulin resistance, or metabolic syndrome. Often results for multiple POPs are reported in the same study. Because of time constraints at the workshop, breakout group participants focused on diabetes outcomes, although findings related to glucose homeostasis, insulin resistance, and metabolic syndrome will be summarized in supplemental materials that accompany the POPs breakout group report. The breakout group developed a quality rating for each study based primarily on the methods used to classify or measure exposure, and the diagnostic used to ascertain diabetes status. Studies received a lower rating if the diagnoses of diabetes came from death certificates, if diabetes was self--reported, if exposure was self-reported, or if exposure was not clearly measured. The breakout group then used the Meta Data Viewer program to assess patterns of association between various POPs chemicals or chemical classes and diabetes (Boyles et al. 2011).

**Figure 3:**
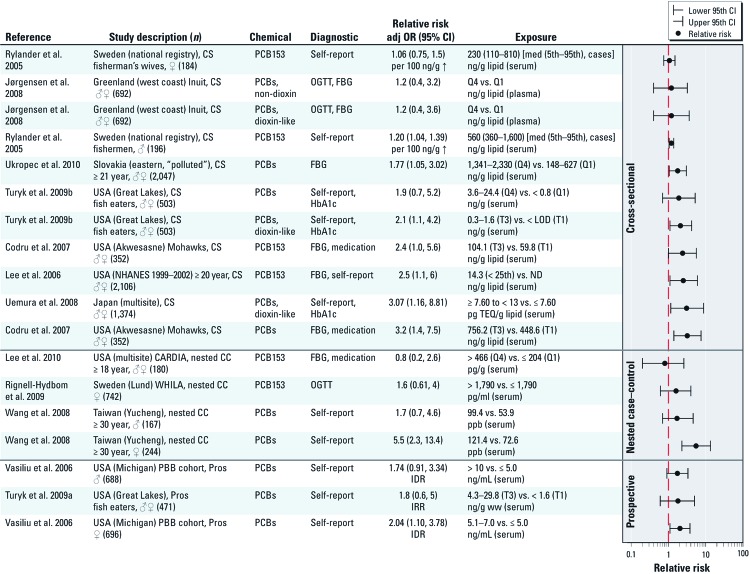
Association between PCBs and diabetes. Studies are grouped by study design and then sorted alphabetically by first author within each study design category. Abbreviations: CARDIA, Coronary Artery Risk Development in Young Adults; CS, cross-sectional; FBG, fasting blood glucose; IDR, incidence density ratio; IRR, incidence rate ratio; LOD, limit of detection; med, median; MI, Michigan; ND, not detected; NHANES, National Health and Nutrition Examination Survey; PBB, polybrominated biphenyl; Q, quartile; T, tertile; TEQ, toxic equivalency; WHILA, Women’s Health in the Lund Area; ww, wet weight.

The group concluded that there is evidence for a positive association of diabetes with certain organochlorine POPs. Initial data mining indicated the strongest associations of diabetes with *trans*-nonachlor, DDT (dichloro-diphenyltrichloroethane)/DDE (dichloro-diphenyldichloroethylene)/DDD (dichloro-chlorophenylethane), and dioxins/dioxin-like chemicals, including polychlorinated biphenyl (PCBs; Figure 3). In no case was the body of data considered sufficient to establish causality. The very strong exposure correlations among some POPs [correlation coefficients of 0.50–0.90 (Lee et al. 2006)] make it difficult to identify individual POPs as potential causal agents.

*Peroxisome proliferator–activated receptor (PPAR) activators (organotins and phthalates).* Organotins and phthalates were considered together in a breakout group session because these compounds both interact with PPARs. The PPARs are intimately involved in the regu-lation of adipocyte differentiation, production of adipokines, insulin responsiveness, and other biological processes related to glucose and lipid regulation (Janesick and Blumberg 2011; Kahn and McGraw 2010; Li et al. 2011; Wang 2010). In addition, there is the potential for coexposures to these two chemical classes because both are commonly used as plasticizers in PVC (polyvinylchloride) plastics. The extent and magnitude of exposure are assumed to be higher for phthalates than for organotins, but exposure to organotins is not well characterized (Kannan et al. 2010).

The pattern of stimulatory activity for specific PPAR receptor subtypes varies between the organotins [primarily tributyltin (TBT)] and individual phthalates, with the organotins appearing to have a stronger mechanistic profile for inducing “obesogenic” effects. The organotins are potent agonists for PPARγ as well as retinoid X receptor-α (RXRα), two receptors known to promote adipocyte differentiation *in vitro* when activated (Grun et al. 2006; Hiromori et al. 2009; Inadera and Shimomura 2005; Kanayama et al. 2005; le Maire et al. 2009; Nakanishi et al. 2005; Nishikawa et al. 2004). Because PPARγ and RXRα heterodimerize, organotins stimulate both parts of the heterodimer complex.

The phthalates are less potent activators of PPARγ than are organotins, with agonist activity occurring at concentrations 1,000 times higher (~ 10–100 µM vs. ~ 10–100⊇nM), and phthalates have not been identified as agonists for RXRα. In contrast to the organotins, the phthalates are more potent agonists for PPARα than for PPARγ. The organotins are not considered activators of PPARα (Blumberg B, personal communication, 28 November 2010). In rodent models, PPARα appears to mediate high-dose di(2-ethylhexyl) phthalate (DEHP)-induced body weight loss, but its role in regulating adipogenesis is less clear (Wang 2010).

Organotins. No epidemiological studies of organotin exposure and obesity or diabetes were identified during the literature search. There are poisoning incident reports, mostly in workers involved in applying the compounds for pesticide use, that describe incidents of hyperglycemia and/or glycosuria (Colosio et al. 1991; Manzo et al. 1981; reviewed by National Institute for Occupational Safety and Health 1976). Recent animal and mecha-nistic studies report stimulatory effects of TBT on adipocyte differentiation (*in vitro* and *in vivo*) and increased amount of fat tissue (i.e., larger epididymal fat pads) in adult animals exposed to TBT during fetal life (Grun and Blumberg 2006; Hiromori et al. 2009; Inadera and Shimomura 2005; Kanayama et al. 2005; Kirchner et al. 2010; Nakanishi et al. 2005). *In vitro* effects of TBT include increased lipid accumulation in adipocytes and increased differentiation of multipotent stromal stem cells into adipocytes (Kirchner et al. 2010). Although the organotin “obesogen” literature is relatively new, with few studies, the quality of the existing experimental studies was considered high by the breakout group.

Phthalates. Three cross-sectional human studies of exposure to phthalates were discussed by the breakout group (Boas et al. 2010; Hatch et al. 2008; Stahlhut et al. 2007). These studies reported some positive associations but did not provide sufficient evidence to conclude there is an association with diabetes or obesity. Therefore, findings suggesting the possibility of sex differences in associations and different effects of individual phthalates were considered preliminary. In these studies, the urinary phthalate metabolite monoethyl phthalate was the phthalate metabolite most often associated with higher body mass index (BMI) (Hatch et al. 2008), waist circumference (Stahlhut et al. 2007), or HOMA (Stahlhut et al. 2007). Mono-2-ethylhexyl phthalate was associated with decreased BMI in females > 12 years of age (Hatch et al. 2008).

Understanding differences in PPARα activity between humans and rodents is important with respect to understanding potential effects of phthalates on body weight and metabolic end points. Phthalate monoester metabolite concentrations required to activate human PPARα are two to three times higher than the concentrations required to activate mouse PPARα, and the maximum-fold induction is less for human PPARα than for mouse PPARα (Bility et al. 2004; Hurst and Waxman 2003; Maloney and Waxman 1999). Animals treated with relatively high doses of phthalates such as DEHP typically display decreased body weight and fat mass (Itsuki-Yoneda et al. 2007; Sakurai et al. 1978). These effects were not observed in PPARα-knockout mice (Feige et al. 2010), which suggests they are largely mediated via the PPARα agonist activities of DEHP metabolites (Feige et al. 2010; Martinelli et al. 2010). However, when the normal mouse PPARα gene was replaced with the human PPARα gene, mice treated with DEHP gained weight and had increased epididymal white adipose mass compared with wild-type animals (Feige et al. 2010). PPARγ activity is similar in rodents and humans, but stronger PPARα activity in mice compared with humans may mask effects mediated through PPARγ.

*BPA.* Overall, this breakout group concluded that the existing data, primarily based on animal and *in vitro* studies, are suggestive of an effect of BPA on glucose homeostasis, insulin release, cellular signaling in pancreatic β cells, and adipogenesis (Alonso-Magdalena et al. 2010; Miyawaki et al. 2007; Ryan et al. 2010; Somm et al. 2009). The existing human data on BPA and diabetes (Lang et al. 2008; Melzer et al. 2010) available at the time of the workshop were considered too limited to draw meaningful conclusions. Similarly, data were insufficient to evaluate BPA as a potential risk factor for childhood obesity: Only one pilot study was available at the time of the workshop (Wolff et al. 2008).

It was not possible to reach clear conclusions about BPA and obesity from the existing animal data. Although several studies report body weight gain after developmental exposure, the overall pattern across studies is inconsistent. However, breakout group participants emphasized that body weight is not considered a good measure of obesity in rodents and noted that only a few studies have assessed obesity using the preferred metrics such as fat mass, fat pad weight, and cell adipose tissue cellularity. There is inconsistency in the *in vivo* findings that may relate to differences in experimental designsuch as differences in diet, route of administration, and species/strain. Understanding the basis for these inconsistencies was considered a research priority. The group also noted that the mechanisms of BPA action are not fully understood but extend beyond its activity as an estrogen receptor agonist. A number of *in vitro* findings suggest interactions with other receptor systems involved in metabolic regulation (Wetherill et al. 2007), including anti-androgen effects at low concentrations and high binding affinity for estrogen-related receptor-γ (Takayanagi et al. 2006).

*Pesticides.* The pesticide breakout group concluded the epidemiological, animal, and mechanistic data support the biological plausibility that exposure to multiple classes of pesticides may affect risk factors for diabetes and obesity, although many significant data gaps remain. Some active ingredients of pesticides, and of insecticides in particular, affect neurotransmitter and/or ion channel systems that are also involved in regulating pancreatic function, including acetylcholine (e.g., organophosphate, carbamate, neonicotinoids), sodium channels (e.g., pyrethroids), γ-aminobutyric acid (e.g., organochlorine), catecholamine (e.g., amidine/-formamidine), and mitochondrial function (e.g., rotenone). This raises the possibility that these compounds might affect glucose homeostasis, at least at dose levels where they are effective as pesticides (Franklin and Wollheim 2004; Satin and Kinard 1998). Much less research has focused on whether pesticides have activities that might affect adiposity or other components for metabolic syndrome.

Case reports of hyperglycemia have been reported after poisoning incidents with a variety of pesticides, perhaps best documented for organophosphates (Agency for Toxic Substances and Disease Registry 1997; Sungur and Guven 2001) and the formamidine insecticide amitraz (Caksen et al. 2003; Elinav et al. 2005; Ertekin et al. 2002; Kennel et al. 1996; Ulukaya et al. 2001; Yilmaz and Yildizdas 2003). Type 1 diabetes is a recognized complication after accidental poisoning with the banned rodenticide Vacor (Gallanosa et al. 1981; Karam et al. 1980; Miller et al. 1978; Mindel 1986; Peters et al. 1981; Pont et al. 1979; Prosser and Karam 1978; Yoon 1990). Vacor is structurally similar to strepto-zotocin, a compound widely used to induce experimental diabetes in animals. With the exception of studies of persistent organo-chlorine pesticides such as DDT/DDE or *trans*-nonachlor, there are very few cohort studies of other pesticides and health conditions related to diabetes, metabolic syndrome, or adiposity.

There have been numerous reports of intoxication with organophosphate insecticides on blood glucose in laboratory animals, generally finding hyperglycemia at high dose levels (see reviews by Karami-Mohajeri and Abdollahi 2010; Rahimi and Abdollahi 2007). Recently, the focus of investigations has shifted toward studies designed to understand the consequences of developmental exposure to lower doses of organophosphates, and the long-term effects of these exposures on metabolic dysfunction, diabetes, and obesity later in life (Adigun et al. 2010a, 2010b, 2010c; Icenogle et al. 2004; Lassiter et al. 2008, 2010; Levin et al. 2002; Roegge et al. 2008; Slotkin et al. 2005, 2009; reviewed by Slotkin 2010). The general findings are that early-life exposure to otherwise subtoxic levels of organophosphates results in pre-diabetes, abnormalities of lipid metabolism, and promotion of obesity in response to increased dietary fat.

The EPA Toxicity Reference Database (ToxRefDB; U.S. EPA 2011b), was also used as a resource for the pesticide breakout group. The current version of the ToxRefDB contains detailed study and effect information on 474 chemicals, primarily the data-rich pesticide active ingredients. Many of these studies were conducted for regulatory purposes and are not available in the peer-reviewed literature. ToxRefDB was queried for chemicals that caused increased body weight (or body weight gain), increased blood glucose, and pancreatic effects, including changes in mass, adenomas, atrophy, congestion, hyperplasia, hypertrophy, inflammation, fatty change, degeneration, and cellular infiltration. Approximately 100 chemicals were causes of at least one of these effects (see NTP 2011b, appendix B). Six of the studies identified increased body weight as a result of treatment with several organophosphates, including two separate studies for fenthion, one conducted in rats and the other in mice (Table 1). Several sulfonylurea herbicides and imidazole fungicides were also identified by the ToxRefDB search. These pesticides belong to the same general chemical class as agents used to manage type 2 diabetes or that are being investigated as potential therapeutic agents.

**Table 1:** Selected results from ToxRefDB search for chemicals that caused increased body weight, increased blood glucose, or pancreatic effects.

Doses tested (mg/kg-day)			Effect doses (mg/kg-day)				
			Chemical class/ Chemical name (CASR number)	↑ Body weight	Pancreatic pathology or neoplasia
Study design	Lowest	Highest				↑ Glucose	Reference
Imidazole														
Imazalil (35554-44-0)		SUB, rat, feed		1.25		60				3.75				Lina et al. 1983
Imazalil (35554-44-0)		CHR, mouse, feed		6.67		110						88		Verstraeten 1993
Triflumizole (68694-11-1)		CHR, rat, feed		3.5		77						59.4		Broadmeadow et al. 1984
Inorganic														
Cyanamide (420-04-2)		CHR, rat, gavage/ intubation		1		7.5		7.5						Osheroff 1991
Organophosphate														
Azamethiphos (35575-96-3)		CHR, mouse, feed		6.2		614.3						614		Goodyer 1987
Dichlorvos (62-73-7)		CHR, rat, gavage/ intubation		4		8						8		Chan 1987
Dimethoate (60-51-5)		CHR, rat, feed		0.05		5						1.25		Squire 1988
Disulfoton (298-04-4)		CHR, mouse, feed		0.15		2.4		2.4						Mobay Chemical Corporation 1983
Fenthion (55-38-9)		CHR, mouse, feed		0.03		10.63		1.95						Leser and Suberg 1990
Fenthion (55-38-9)		MGR, rat, feed		0.05		5		5						Kowalski et al. 1989
Malathion (121-75-5)		CHR, rat, feed		4		868						29		Daly 1996
Parathion-methyl (298-00-0)		CHR, mouse, feed		0.2		13.7		9.2						Eiben 1991
Propetamphos (31218-83-4)		CHR, rat, feed		0.376		7.602						0.689, 7.6		Luginbuehl 1980
Tebupirimfos (96182-53-5)		CHR, mouse, feed		0.52		43.57		38.8		38.8				Eiben 1990
Tebupirimfos (96182-53-5)		SUB, rat, feed		0.2		4.9				0.4				Eiben 1989
Tribufos (78-48-8)		CHR, mouse, feed		1.64		63.04		48						Hayes 1989
Sulfonylurea														
Oxasulfuron (144651-06-9)		CHR, rat, feed		0.84		871						425		Pettersen and Morrissey 1996
Sulfosulfuron (141776-32-1)		CHR, rat, feed		2.4		1296.5						244		Naylor and Ruecker 1997
Triasulfuron (82097-50-5)		SUB, rat, feed		10		1,000				1,000				Tai 1985
Tribenuron-methyl (101200-48-0)		CHR, rat, feed		1.25		62.5						62.5		Tobia 1987
Abbreviations: CASR, Chemical Abstracts Service Registry; CHR, chronic; MGR, multigenerational; SUB, subchronic. The complete list can be found online (NTP 2011b; see appendix B in “Draft Literature Review Documents” for pesticides).														

## Use of Tox21 HTS to Identify Substances of Potential Interest

Consideration of data from the Tox21 HTS Initiative played a prominent role in the workshop. Tox21 is a collaborative program between the U.S. EPA, NIEHS/NTP, NIH Chemical Genomics Center, and U.S. Food and Drug Administration (FDA) designed to research, develop, validate, and translate innovative chemical testing methods that characterize toxicity pathways (NTP 2011a). Data from phase I of ToxCast™, U.S. EPA’s contribution to Tox21 (U.S. EPA 2011a), were used to help determine the biological plausibility of reported effects and to identify chemicals that may interact with relevant mechanistic targets but have not been assessed for effects related to diabetes or obesity. In general, the ToxCast™ data often aligned with mechanistic findings in the peer-reviewed literature. For example, the organotin fentin was identified in ToxCast™ as a target for PPARγ at a relatively low concentration (U.S. EPA 2011a; search for “fentin”). Amitraz, a formamidine insecticide, is an α2-adrenoreceptor agonist (Chen and Hsu 1994; Hugnet et al. 1996; Smith et al. 1990), and this activity was identified in ToxCast™ (U.S. EPA 2011a; search for “amitraz”).

Many of the pesticides identified from ToxRefDB as causes of increased body weight, increased blood glucose, or pancreatic effects were also screened in phase I of ToxCast™, providing clues regarding potential mechanisms that may underlie the *in vivo* effects. Preliminary analysis of these results indicates that many pesticides have HTS “hits” that are unrelated to their classic pesticide mechanism of action but may be relevant to biological processes relevant to glucose homeostasis, insulin sensitivity, adipocyte differentiation, and lipid metabolism. However, the chemicals or chemical classes that have been most strongly or consistently associated with diabetes or obesity in humans (*trans*-nonachlor, 2,3,7,8-tetrachlorodibenzo-*p*-dioxin, DDT/DDE/DDD, PCBs, arsenic, nicotine) have not yet been screened in ToxCast™.

Data from phase I of ToxCast™ were also used to develop testable predictions of which chemicals might perturb biological processes related to diabetes and obesity. In brief, workshop participants identified relevant HTS targets for several biological processes related to diabetes and obesity (insulin signaling, islet cell function, adipocyte differentiation, and feeding behavior in *Caenorhabditis elegans*). The 309 chemicals tested in phase I of ToxCast™, primarily pesticide active ingredients, were then screened against these targets to identify a set of chemicals predicted to perturb these processes and others predicted to have no effect. As a follow-up activity, the NTP is initiating a targeted testing activity for a set of predicted “positives” and “negatives” using more physiologically based *in vitro* model systems. Experts also suggested biological assay targets that could be added to Tox21 to improve the ability to identify chemicals that may perturb metabolic processes.

## Conclusions, Research Recommendations, and Next Steps

Overall, the workshop review of the existing litera-ture supports the plausibility of the “obeso-gen” hypothesis, as well as linkages between type 2 diabetes and exposures to certain chemical classes. A review of the literature indicates very little research has been directed toward understanding associations between environmental exposures and type 1 diabetes. This was considered a critical data gap. Many research questions remain, and an important goal of this workshop was to identify data gaps to stimulate focused research to move the field forward. The research recommendations included suggestions for the most appropriate end points to evaluate in human, animal, and mechanistic studies of diabetes and obesity (Tables 2–4). All of the breakout groups highlighted the importance of using clinically accepted measures of diabetes and overweight/obesity in the epidemiological studies (Table 4). Understanding more about the different phenotypes of obesity will require more sophisticated measurement methods because the distribution of adipose tissue can vary among individuals with the same BMI and waist circumference. Another series of recommendations was to elucidate the role(s) of effect modifiers, confounding factors, and specific genetic contributions in humans and animal models used to study these diseases (Table 3).

**Table 2:** Research recommendations for health outcome assessment measures.

Humans			Animal and mechanistic models		
Diabetes	Obesity	Diabetes	Obesity
Use accepted diagnostic criteria Other relevant end points: plasma insulin, insulin tolerance, insulin resistance, and β-cell function (i.e., HOMA) Not recommended: glucosuria and documentation of diabetes only through death certificates		Use accepted diagnostic criteria Other relevant end points: adipose deposition and distribution (MRI, DXA, and NMR), bone density end points for PPARγ-active compounds		Use fasting and fed blood glucose and insulin, GTT, ITT, insulin resistance (i.e., HOMA), insulin signaling pathways (peripheral and β cell) Not recommended: glucosuria		Measure adipose deposition and distribution (fat mass, fat pads), adipocyte cellularity, feeding behavior, energy balance, brain and peripheral inflammation, neurohumural signaling, body weight, body length, bone density end points for PPARγ-active compounds


**Table 3:** Factors to consider for interactions, effect modification, and potential confounding.

Humans	Animal and mechanistic models
Age, BMI, sex, physical activity, socioeconomic variables, food consumption/dietary intake, smoking status, concurrent medication (e.g., statins, metformin), significant exposures to other agents, measure of health status including kidney function and recent weight changes Developmental studies: maternal BMI, maternal weight gain during pregnancy, maternal age, maternal diabetes (gestational or type 2), maternal diet, parental smoking, infant diet (breast-feeding vs. formula feeding), introduction of food during nursing period, childhood diet, childhood physical activity		Postnatal diet and dietary factors, including high-fat diet challenges Animal models: consider species and strain differences (e.g., chemical-specific pharmacokinetics, PPARα and other receptors in rodent and human), disease state of interest (type 1 or type 2 diabetes), genetic diversity of the model


**Table 4:** Diagnostic criteria for human studies (BMJ Group 2011).

Diabetes mellitus, type 2	Prediabetes	Overweight/obese adults	Overweight/obese children
Random PG level ≥ 11.1 mmol/L (200 mg/dL) in the presence of symptoms of hyperglycemia FPG glucose ≥ 7.0 mmol/L (126 mg/dL) 2-hr PG level ≥ 11.1 mmol/L (200 mg/dL) during OGTT with 75 g oral glucose load HbA1c ≥ 6.5%		FPG 5.6–6.9 mmol/L (100–125 mg/dL) 2-hr postload glucose after 75 g oral glucose of 7.8–11.0 mmol/L (140–199 mg/dL) HbA1c of 5.7–6.4% indicates prediabetes or high risk of future diabetes		BMI Overweight: BMI 25.0–29.9 Obese: BMI 30.0–39.9 Extremely or morbidly obese: BMI ≥ 40.0 Waist circumference Men, > 102 cm; Women, > 88 cm Waist:height ratio Men: ideal, 0.9; increased risk, > 1.0 Women: ideal, 0.7; increased risk, > 0.85		BMI Overweight: BMI 85th to 94th percentile (children > 2 years of age) Obese: BMI ≥ 95th percentile (children > 2 years of age) or weight ≥ 95th percentile for height (children < 2 years of age) Other diagnostic factors Increased waist circumference, increased waist:hip ratio, increased skinfold thickness
Abbreviations: DXA, dual-emission X-ray absorptiometry; FPG, fasting plasma glucose; GTT, glucose tolerance test; ITT, insulin tolerance test; MRI, magnetic resonance imaging; NMR, nuclear magnetic resonance; PG, plasma glucose.						

Many of the research gaps were not unique to the field of diabetes/obesity research. The workshop noted *a*) deficiencies in data on human exposures to many of the chemicals examined, *b*) the need for better biomarkers of exposure that may be related mechanistically to the disease end points, *c*) the need for a better understanding of the basic biology of adipocytes, β cells, and neural circuits that regulate feeding behavior in healthy and disease states, and *d*) the need for an appreciation of how the biology that controls body weight and metabolic set points changes with life stage. A number of the breakout groups noted the need to consider non-monotonic dose–-response relationships for environmental influences on obesity and diabetes. Also, there is a need to consider coexposures between environmental chemicals and consumption of high-calorie, high-carbohydrate, and/or high-fat diets. Finally, workshop participants found the incorporation of HTS information from the Tox21 program to be an intriguing and useful way of improving our understanding of the similarities and differences in biological actions across classes of chemicals and recommended many specific targets for further assay development to further enhance its utility.

NIEHS has already taken steps to address some of the research needs, recognizing that the work will best be accomplished through the combined efforts of the NTP, the NIEHS Division of Extramural Research and Training (DERT), and the NIEHS Division of Intramural Research. Based on the results of this workshop and the data gaps noted, the DERT released program announcements focused on improving our understanding of the role of environmental exposures in the develop-ment of obesity and diabetes (see NIEHS 2011a, 2011b). The announcements have one receipt date per year for the next 3 years. The NTP is organizing further *in vitro* targeted testing of some of the predictions of chemical effects from the Tox21 screening program and is specifically developing an analytical method to measure organotins in human blood because the lack of exposure data to these compounds was considered a critical research need.

We hope this workshop will stimulate further research to better understand the public health impacts of environmental influences on the increasing international prevalence of diabetes, obesity, and metabolic syndrome. We acknowledge the dedicated efforts of the workshop participants toward achieving this goal.

## Supplemental Material

(1.1 MB) PDF
