# Supplementary material for: Role of Environmental Chemicals in Diabetes and Obesity: A National Toxicology Program Workshop Review
Source: Environ Health Perspect. 2012 Feb 1;120(6):779–89. doi: 10.1289/ehp.1104597 (PMC3385443; doi:10.1289/ehp.1104597)
Supplement: (1.1 MB) PDF [file ehp.1104597.s001.pdf]

SUPPLEMENTAL MATERIAL

Role of Environmental Chemicals in Diabetes and Obesity: A National Toxicology Program Workshop Report

Kristina A. Thayer, Jerrold J. Heindel, John R. Bucher, Michael A. Gallo

6 March 2012: The last line of text on page 3 ["...leptin OR resistin OR adiponectin*)) AND (publisher[sb] OR "in process"[sb])"] was missing from the Supplemental Material file posted on 1 February 2012. The text is included in this version of the file.

LITERATURE SEARCH STRATEGY

MeSH: (("amitraz"[Substance Name] OR "arsenic"[Mesh] OR "Polychlorinated Biphenyls"[Mesh] OR "Hydrocarbons, Chlorinated"[Mesh] OR "Dioxins"[Mesh] OR "Halogenated Diphenyl Ethers"[Mesh] OR "Polybrominated Biphenyls"[Mesh] OR "perfluorooctane sulfonic acid"[Substance Name] OR "perfluorooctanoic acid"[Substance Name] OR "Carbon Tetrachloride"[Mesh] OR "Cadmium"[Mesh] OR "Organothiophosphorus Compounds"[Mesh] OR "phthalic acid"[Substance Name] OR "Phthalic Acids"[Mesh] OR phthalate* OR "Organotin Compounds"[Mesh] OR "Atrazine"[Mesh] OR "bisphenol A"[Substance Name] OR "Nicotine"[Mesh] OR "Genistein"[Mesh]) AND (("obesity"[mh] OR "body mass index"[mh] OR "weight gain"[mh] OR "adipogenesis"[mh] OR "adipose tissue"[mh] OR "adipokines"[mh] OR "adiponectin"[mh] OR "leptin"[mh] OR resistin[mh]) OR ("diabetes mellitus"[mh] OR "glucose metabolism disorders"[mh] OR "insulin"[mh] OR "insulin resistance"[mh] OR "blood glucose"[mh] OR "islets of langerhans"[mh])) AND (("obesity"[mh] OR "body mass index"[mh] OR "weight gain"[mh] OR "adipogenesis"[mh] OR "adipose tissue"[mh] OR "adipokines"[mh] OR " adiponectin"[mh] OR "leptin"[mh] OR resistin[mh]) OR ("diabetes mellitus"[mh] OR "glucose metabolism disorders"[mh] OR "insulin"[mh] OR "insulin resistance"[mh] OR "blood glucose"[mh] OR "islets of langerhans"[mh])))

Keyword: ((amitraz OR arsenic OR "Polychlorinated Biphenyls" OR "chlorinated hydrocarbons" OR aldrin OR "carbon tetrachloride" OR chlordane OR chlordecone OR chlorobenzene* OR hexachlorobenzene OR chloroform OR ddt OR dichlorodiphenyltrichloroethane OR dichloroacetate OR "dichlorodiphenyl dichloroethylene" OR dichlorodiphenyldichloroethane OR dichloroethylenes OR dieldrin OR endrin OR "ethyl chloride" OR "ethylene dichlorides" OR heptachlor OR lindane OR hexachlorocyclohexane OR methoxychlor OR "methyl chloride" OR "methylene chloride" OR mirex

OR mitotane OR "picryl chloride" OR polychloroterphenyl OR tetrachloroethylene OR toxaphene OR trichloroepoxypropane OR trichloroethane* OR trichloroethylene OR "vinyl chloride" OR "Dioxins" OR TCDD OR "Halogenated Diphenyl Ethers" OR "diphenyl ethers" OR PBDE* OR PCDE* OR "Polybrominated Biphenyls" OR "polybrominated biphenyls" OR Polybromobiphenyl* OR "polychlorinated b iphenyls" OR Polychlorobiphenyl OR PCB OR "perfluorooctane sulfonic acid" OR "perfluorooctane sulfonic acid" OR pfosa OR 1763-23-1 OR "perfluorooctane sulfonate" OR "perfluorooctanoic acid" OR 335-67-1 OR "perfluorooctanoic acid" OR PFOA OR "pentadecafluorooctanoic acid" OR "perfluorooctanoyl chloride" OR "sodium perfluorooctanoate" OR "perfluorinated octanoic acid" OR "Carbon Tetrachloride" OR "Cadmium" OR "Organothiophosphorus Compounds" OR organothiophosphorus OR amifostine OR azinphosmethyl OR chlorpyrifos OR coumaphos OR cystaphos OR diazinon OR dimethoate OR disulfoton OR "echothiophate iodide" OR fenitrothion OR fenthion OR fonofos OR leptophos OR malathion OR parathion OR "phenylphosphonothioic acid" OR phorate OR phosmet OR temefos OR "thiophosphoric acid" OR thiotepa OR "phthalic acid" OR phthalate* OR "phthalic acids" OR "dibutyl phthalate" OR "diethylhexyl phthalate" OR "o-phthalaldehyde" OR "phthalic anhydrides" OR phthalimides OR thalidomide OR organotin OR "di -n-butyltin" OR dibutyltin OR dibutylstannae OR 1002-53-5 OR "triphenyltin chloride" OR 639-58-7 OR tributyltin OR "tri-n-butyltin" OR 688-73-3 OR trialkyltin OR triethyltin OR trimethyltin OR Atrazine OR "bisphenol A" OR Nicotine OR genistein) AND ((diabetes OR "glucose tolerance" OR "glucose intolerance" OR hyperglycemia OR hypoglycemia OR insulin OR "blood glucose" OR "metabolic syndrome" OR "syndrome x" OR "islets of langerhans") OR (obes* OR "body mass index" OR "body fat" OR "weight gain" OR adipos* OR adipogen* OR adipokine* OR leptin OR resistin OR adiponectin*)) AND (publisher[sb] OR "in process"[sb])
